# Mycorrhizal symbiosis and the nitrogen nutrition of forest trees

**DOI:** 10.1007/s00253-024-13298-w

**Published:** 2024-09-09

**Authors:** Rodica Pena, Mark Tibbett

**Affiliations:** 1 Department of Sustainable Land Management, School of Agriculture, Policy and Development, University of Reading, Reading, UK; 2 Department of Silviculture, Transilvania University of Brasov, Brasov, Romania

**Keywords:** Ectomycorrhizal functional traits, Mutualistic spectrum, Decomposition, Nitrogen cycle

## Abstract

**Abstract:**

Terrestrial plants form primarily mutualistic symbiosis with mycorrhizal fungi based on a compatible exchange of solutes between plant and fungal partners. A key attribute of this symbiosis is the acquisition of soil nutrients by the fungus for the benefit of the plant in exchange for a carbon supply to the fungus. The interaction can range from mutualistic to parasitic depending on environmental and physiological contexts. This review considers current knowledge of the functionality of ectomycorrhizal (EM) symbiosis in the mobilisation and acquisition of soil nitrogen (N) in northern hemisphere forest ecosystems, highlighting the functional diversity of the fungi and the variation of symbiotic benefits, including the dynamics of N transfer to the plant. It provides an overview of recent advances in understanding ‘mycorrhizal decomposition’ for N release from organic or mineral-organic forms. Additionally, it emphasises the taxon-specific traits of EM fungi in soil N uptake. While the effects of EM communities on tree N are likely consistent across different communities regardless of species composition, the sink activities of various fungal taxa for tree carbon and N resources drive the dynamic continuum of mutualistic interactions. We posit that ectomycorrhizas contribute in a species-specific but complementary manner to benefit tree N nutrition. Therefore, alterations in diversity may impact fungal-plant resource exchange and, ultimately, the role of ectomycorrhizas in tree N nutrition. Understanding the dynamics of EM functions along the mutualism-parasitism continuum in forest ecosystems is essential for the effective management of ecosystem restoration and resilience amidst climate change.

**Key points:**

*• Mycorrhizal symbiosis spans a continuum from invested to appropriated benefits.*

*• Ectomycorrhizal fungal communities exhibit a high functional diversity.*

*• Tree nitrogen nutrition benefits from the diversity of ectomycorrhizal fungi.*

**Graphical abstract:**

**Figure:**
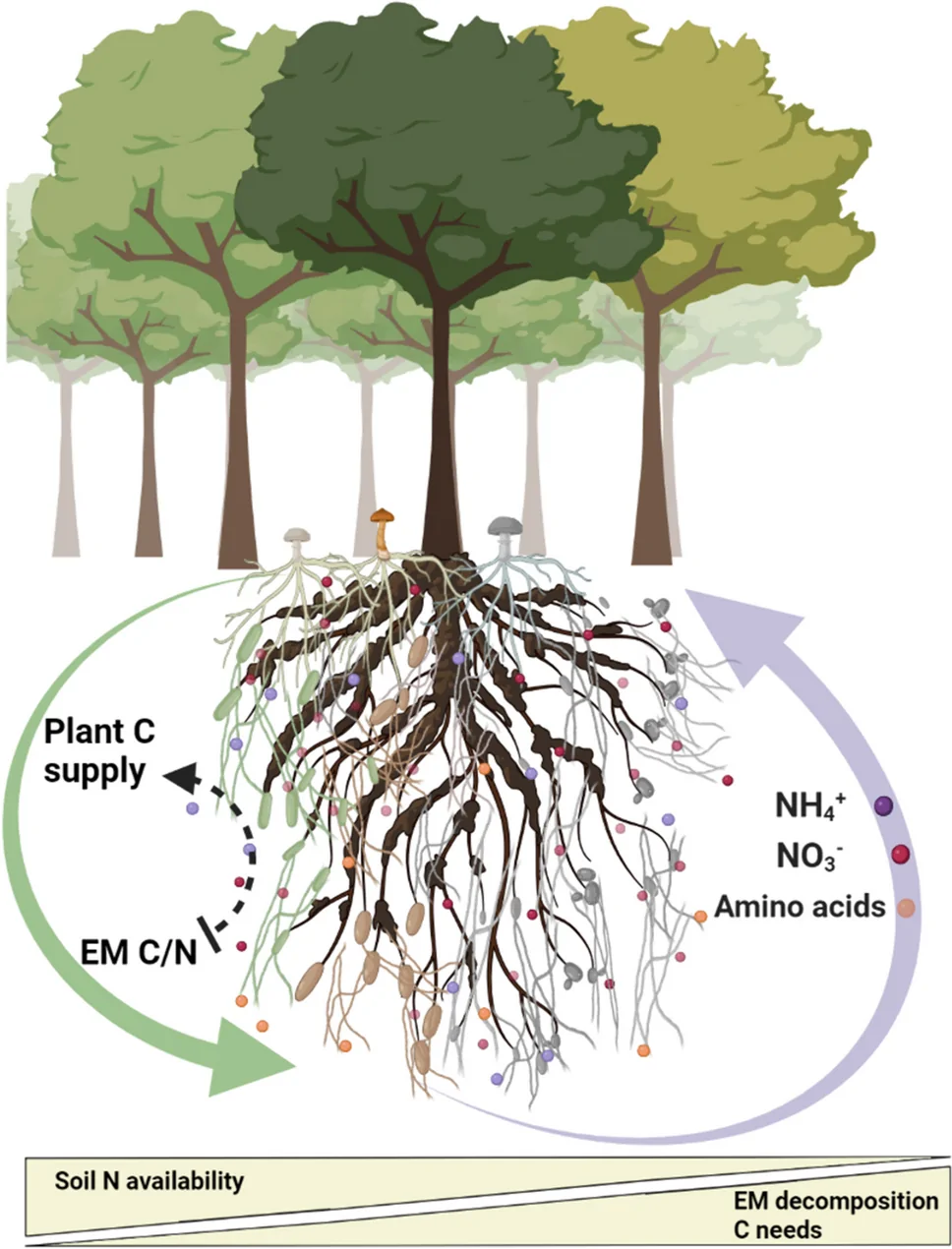

## Introduction

The holobiont concept, in which plants and their associated microbes are viewed not as independent entities but as a cohesive evolutionary unit, emphasises the vital roles that bacteria, fungi, and other microorganisms play in plant growth, health, and adaptation to various environments (Theis et al. 2016; Uroz et al. 2019). Terrestrial plants associate with mycorrhizal fungi to acquire nutrients (Moreau et al. 2019; Sun et al. 2021). They form a symbiotic relationship based on a nutritional exchange between the partners in a *quid pro quo* (‘giving and taking’) manner (Almario et al. 2022). Isotope tracing experiments have long demonstrated that there is a transfer of nutrients between partners (Finlay et al. 1989; Le Tacon et al. 2015; Schreider et al. 2022; Khokon et al. 2023; Pena et al. 2023). The mycorrhizal fungus obtains carbon (C) from the plant, which, in exchange, receives soil nutrients, mainly nitrogen (N) and phosphorus (P) from the fungus (Smith and Read 2010; Sun et al. 2021; Martin and van der Heijden 2024).

**Fig. 1:**
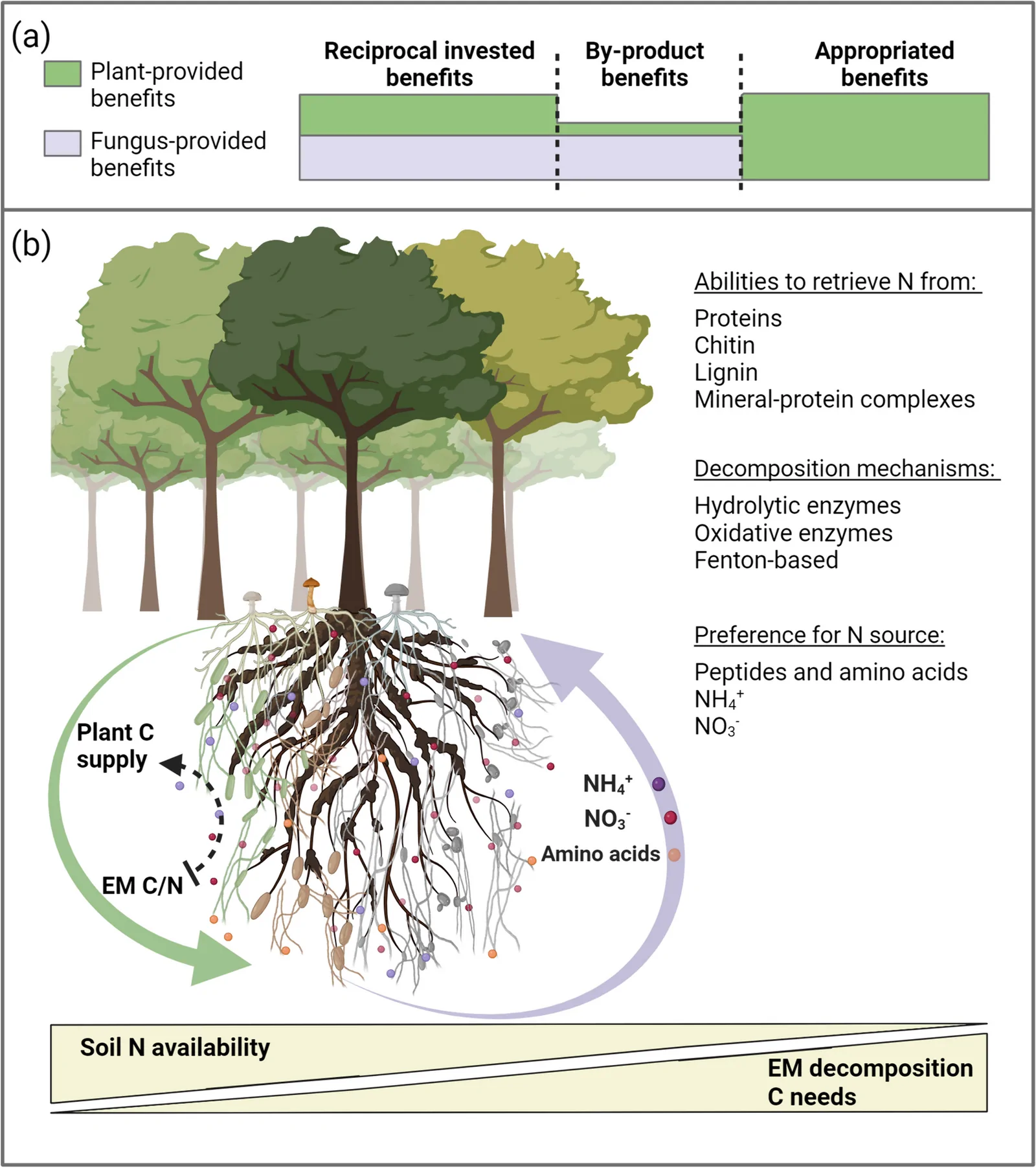
**a** Benefits of mutualistic ectomycorrhizal symbiosis: reciprocal invested benefits occur when both partners actively invest in each other; by-product benefits arise when one organism (the tree) incidentally benefits the other (the fungus); and appropriated benefits happen when one partner (the fungus) exploits the resources of the other (the plant). **b** The contribution of ectomycorrhizal (EM) fungi to tree nitrogen (N) nutrition in forest ecosystems: ectomycorrhizas formed with different fungal taxa vary in their abilities to retrieve N from diverse substrates, their mechanisms of organic matter decomposition and their preferences for specific N sources. The allocation of plant carbon (C) to different ectomycorrhizas is influenced by their C/N ratio. When soil N availability decreases, EM fungi may enhance decomposition activities to access N that requires higher C needs. Under severe N limitation, the mutualistic symbiosis may shift towards appropriated N benefits. The fungus optimises its N use efficiency to lower the C/N ratio, thereby securing more C from the plant without transferring the N to the plant. Created with BioRender.com

Mycorrhizal symbiosis is based on reciprocal invested benefits. Both partners invest and benefit from the symbiosis, and the benefit obtained from interaction exceeds the cost of the investment (Connor 1995). While diverse dynamics ranging from mutualism to parasitism can occur in certain contexts, such as early seedling development, high fertilisation conditions, or mismatched plant and fungal genotypes (Johnson et al. 1997), mycorrhizal symbiosis remains at the mutualistic end of the spectrum (Fig. 1a). It can sometimes be regarded as pseudo-reciprocity, where one partner does not invest directly but provides a by-product benefit to the other partner, such as when C is an excess resource for the plant (Corrêa et al. 2012), or when plants invest in the formation of new root tips, which are also utilised by mycorrhizal fungi (Ruotsalainen et al. 2022). In the case of appropriated (i.e. purloined) benefits (Connor 1995), the fungal partner may exploit the C resources of the plant without any direct investment (e.g. providing N). However, despite this parasitic interaction from the perspective of N nutrition, the fungus may also produce other benefits for the host plant (e.g. providing P) that exceed the costs of the appropriated benefits, maintaining a mutualistic symbiosis.

Of all vascular plant species on earth, approximately 2% (*ca*. 8500 species) form EM symbiosis and most temperate and boreal tree species, accounting for about 60% of tree stems on earth, may associate with some of the > 20,000 fungal species to form ectomycorrhizal (EM) symbioses (Brundrett and Tedersoo 2018; Steidinger et al. 2019). In this symbiosis, plant and fungal cells are reprogrammed to form a specialised root structure, the ectomycorrhiza, which facilitates the uptake and transfer of nutrients (Hacquard et al. 2013; Garcia et al. 2016; Nehls and Plassard 2018). The EM fungus forms a sheath around the colonised root tip (i.e. mantle) from which the mycelium extends bidirectionally into the root apoplastic space and surrounding soil. In the root, the fungus wraps around each cortical cell to create a ‘Hartig net’ (named after Theodor Hartig, who in 1842 described the net but wrongly interpreted it to be plant tissue, Hacskaylo 2017; Sportes et al. 2021). The Hartig net forms the symbiotic interface, where carbohydrate and nutrient exchange between plant and fungal cells take place via transporters. In the soil, the extraradical mycelium extends over varying distances, displaying a diverse range of morphologies depending on the fungal species. The EM extraradical mycelium has been classified into four main soil exploration types based on the extent, differentiation, and quantity of hyphae emanating from the mantle (Agerer 2001). The ‘contact’ exploration type features a smooth mantle, with only a few emanating hyphae primarily involved in nutrient exchange directly at the root interface. The ‘short-distance’ exploration type extends hyphae a short distance from the root surface, forming a compact network that enhances nutrient acquisition within the root depletion zone. The ‘medium-’ to ‘long-distance’ exploration types have hyphae that extend further, forming extensive networks, sometimes involving rhizomorphs that can spread considerably, accessing nutrients from a larger volume of soil beyond the root depletion zone. In natural forests, nearly all fine absorptive roots develop into ectomycorrhizas (Pena et al. 2010, 2017), making EM roots the primary nutrient-absorbing structures for these trees (Martin and van der Heijden 2024). They play a crucial role in major ecosystem functions by influencing tree nutrient acquisition under nutrient-limited conditions (Smith and Read 2010; Henriksson et al. 2021) and contributing to organic matter decomposition (Lindahl and Tunlid 2015; Fernandez and Kennedy 2016; Zak et al. 2019; Lindahl et al. 2021). Additionally, they aid in soil C stabilisation through their recalcitrant mycelium (Clemmensen et al. 2015; Fernandez and Kennedy 2018; Maillard et al. 2023a; Hagenbo et al. 2024).

Recent large-scale comparative genomics, coupled with gene expression studies and classical physiological assessments, have revealed significant differences in functional traits among EM taxa (Miyauchi et al. 2020; Khokon et al. 2023; Maillard et al. 2023b; Auer et al. 2024). Although mycorrhizal relationships predominantly remain mutualistic, Frank’s 1885 definition, which asserts that fungi provide host plants with nutrients (Frank 2005), requires refinement. This is necessary to account for functional variation among individual fungal species (Clemmensen et al. 2021; Lebreton et al. 2021; Lindahl et al. 2021) and differing growth conditions (Henriksson et al. 2021). This review synthesises recent advances in our understanding of the dynamics of EM symbiosis in terms of resource acquisition and exchange. It emphasises the role of EM in enhancing tree N nutrition in the large context of varying soil N availability.

Nitrogen is an essential element whose availability regulates primary productivity and organic matter decomposition in terrestrial ecosystems (LeBauer and Treseder 2008; Vitousek et al. 2010). In Northern Hemisphere forests, the low availability of biological N forms is a major factor limiting plant growth (LeBauer and Treseder 2008). In the short term, N availability is driven by the balance between supply and demand (von Sperber et al. 2017). Under low supply, strong plant and microbial demands result in rapid uptake and immobilisation of any available N, aggravating the limitation (von Sperber et al. 2017). Ectomycorrhizal fungi work as nutrient miners and scavengers-carriers (sensu Cao et al. 2024) and are involved in both the supply and demand chains. In the supply chain, they are mobilising N from organic matter as ‘mycorrhizal decomposers’(Lindahl and Tunlid 2015; Lindahl et al. 2021; Martin and van der Heijden 2024). In the demand chain, EM fungi acquire nutrients for the use of themselves and their host trees, becoming some of the strongest N competitors in the forest ecosystems (Wallenda and Read 1999; Pena et al. 2013; Bödeker et al. 2016; Auer et al. 2024).

In the following sections, we summarise the key findings on the EM fungal role in the mobilisation and acquisition of soil N, highlighting the functional diversity of EM fungi (Fig. 1b). Finally, we discuss the variation of mutualism benefits, including the dynamics of N transfer to the plant.

## Contribution of ectomycorrhizas to organic N mobilisation

In most forest soils, up to 95% of N exists in organic form. Before stabilisation in the soil matrix and if not taken up by plants, organic N cycles between microbial biomass and residues, adsorption and desorption from soil mineral particles, and dissolution and precipitation from the soil solution, as described by Bingham and Cotrufo (2016). Organic N enters the soil through plant litter, and root, animal and microbial necromass, as well as dissolved organic N from root exudates and plant litter leachates. The labile N compounds, such as free amino acids or peptides, are rapidly immobilised by microorganisms and plants (Neff et al. 2003; Schimel and Bennett 2004). The remaining organic N, consisting of polymeric structures, is commonly complexed with degradation organic products or mineral particles and must undergo depolymerisation or mineralisation to become available to plants (Nannipieri and Paul 2009; Bingham and Cotrufo 2016; Jilling et al. 2018). In N-poor ecosystems, where organic N-use by plants is common, depolymerisation is the most limiting process in N accessibility (Schimel and Bennett 2004; Näsholm et al. 2009). Saprotrophic fungi and bacteria are recognised as efficient decomposers of organic biopolymers in the soil (López-Mondéjar et al. 2018, 2020). However, some EM basidiomycetes have the ability to secrete extracellular enzymes and low molecular weight (LMW) metabolites to solubilise a range of compounds from simple proteins to chitin, polymerised lignin, and mineral-protein complexes (Tibbett et al. 1999; Shah et al. 2013, 2016; Wang et al. 2020, 2021). In contrast with saprotrophs, which utilise soil organic matter as a source of metabolic C, ‘mycorrhizal decomposition’ results in N mobilisation (Lindahl and Tunlid 2015; Nicolás et al. 2019; Clemmensen et al. 2021). EM fungi use the plant C supply to carry out the co-metabolic decomposition of complexed organic N, releasing N (Hobbie et al. 2013; Lindahl and Tunlid 2015; Nicolás et al. 2019). Shah et al. (2016) propose that during EM evolution, the ancestral decay mechanisms used to obtain C have been changed to obtain N. Given that EM fungi evolved multiple times within different clades of saprotrophs, there are large variations in the genetic potential to decay soil organic matter among EM fungal lineages (Kohler et al. 2015; Miyauchi et al. 2020; Looney et al. 2022; Wu et al. 2022).

The genomes of many EM basidiomycetes retain a reduced set of genes encoding enzymes involved in organic matter decomposition, as compared to saprotrophs. These enzymes are commonly found in decaying mechanisms of white-rot and brown-rot saprotrophic fungi (reviewed in Lebreton et al. 2021). The white-rot fungi mainly degrade the exposed lignocellulose surfaces via extracellular oxidative enzymes, including phenol oxidases such as laccases and class II peroxidases (e.g. lignin peroxidases, manganese peroxidases, or versatile peroxidases) and various hydrolytic carbohydrate-active enzymes (CAZymes) (Janusz et al. 2017). Brown rot fungi evolved from white-rot fungi by losing many of their oxidoreductases and CAZymes as they have developed a less energy-demanding LMW catalytic mechanism based on a Fenton system for generation of hydroxyl radicals (Eastwood 2014; Goodell 2020). These hydroxyl radicals can rapidly depolymerise and then repolymerise lignin in a modified form, which is available for a delayed enzymatic attack (Goodell 2020).

Spectroscopic and genome-wide transcriptome analysis confirmed that EM fungi representing different origins of symbiosis within a white-rot (Agaricales), brown-rot (Boletales), or mixed (Atheliales–Amylocorticales) decomposer clades may retrieve N from soil organic matter extracts using oxidative mechanisms (Shah et al. 2016). Organic matter oxidation is possible as a co-metabolic process (Lindahl and Tunlid 2015) only when C is supplied (i.e. glucose) (Rineau et al. 2013; Shah et al. 2016; Nicolás et al. 2019). Ectomycorrhizal fungi also engage in Fenton-based decomposition of organic matter, similar to brown-rot fungi (Rineau et al. 2012; Shah et al. 2016, 2020). However, the specific mechanisms of Fenton chemistry may vary among different EM fungi (Shah et al. 2020). In an in vitro experiment involving five EM fungal species from different symbiotic origins, all species modified the organic extracts to varying extents, utilising different sets of transcripts (Shah et al. 2016). In a follow-up experiment, Nicolás et al. (2019) demonstrated that the mechanisms of N mobilisation from organic matter extracts differed significantly between *Paxillus involutus* (Boletales) and *Laccaria bicolor* (Agaricales), reflecting their evolutionary origins of brown-rot and litter-decomposing fungus, respectively. *Paxillus involutus* used a time-separated two-step mechanism consisting of oxidation and hydrolysis, whereas *Laccaria bicolor* employed a one-step mechanism that combined the activities of oxidative and hydrolytic enzymes (Nicolás et al. 2019). In both fungi, the availability of C and inorganic N in the culture media governed N mobilisation from the soil organic matter extracts. Decomposition and liberation of organic N decreased under C limitation and started only after the inorganic N (i.e. ammonium) was depleted (Nicolás et al. 2019). In *P. involutus*, oxidation was controlled by N limitation and hydrolysis by C limitation. In *L. bicolor*, the one-step mechanism was initiated by N limitation and sustained during C limitation. Based on the transcriptional pattern, where a gene encoding a sugar transporter and several plant cell wall-degrading enzyme (PCWDE) genes were upregulated, the authors suggested that L. *bicolor* may have the capacity to assimilate C released during organic matter decomposition (Nicolás et al. 2019). This capability is evident, at least when the fungus is not engaged in symbiosis (Martin et al. 2008). The role of N availability in triggering ‘mycorrhizal decomposition’ was also observed in the field, where ammonium addition led to the downregulation of manganese peroxidase genes in the genus *Cortinarius* (Bödeker et al. 2014; Pellitier and Zak 2021; Argiroff et al. 2022). Nevertheless, the control exerted by C availability on ‘mycorrhizal decomposition’ indicates that organic N mobilisation involves energetic costs that are commonly supported by trees on the reciprocal investment benefit mutualism. The benefits provided by the fungus should outweigh the cost of plant C investment to avoid a change leading to appropriated benefits or even parasitism (Näsholm et al. 2013; Baskaran et al. 2017; Ågren et al. 2019).

In contrast with the PCWDE gene copy number, which is higher in the saprotrophic than EM fungal genomes, the proportion of genes encoding for microbial cell wall degrading enzymes (MCWDEs) is similar between EM and saprotrophic fungi (Miyauchi et al. 2020). Recently, Auer et al. (2024) have shown that in situ EM fungi most strongly expressed the genes encoding for enzymes that act on chitin, such as GH18 (chitinases) and GH20 (b-N-acetylglucosaminidases). The ability of EM fungi to access N-containing polymers such as chitin or chitosan has been described for numerous species (Maillard et al. 2023b). Some species, such as *Boletus edulis*, *Imleria badia*, *Suillus luteus*, and *Hebeloma cylindrosporum*, are particularly efficient in mobilising N from chitin (Maillard et al. 2023b). This phenomenon is particularly important as fungal mycelium necromass is a large component of soil organic matter (Awad et al. 2019; Hagenbo et al. 2024).

A large fraction (e.g. 45% in boreal forest soil) of soil organic N exists as proteinaceous compounds associated with mineral particles (Jilling et al. 2018; Kramer and Chadwick 2018). Recent works indicate that EM fungi of different phylogeny and ecology can mobilise N from iron oxide mineral-associated proteins (Wang et al. 2020, 2021; Krumina et al. 2022). They use a proteolytic mechanism based on the formation of enzyme–substrate complexes at the mineral surfaces that enables protein hydrolysis without initial desorption of the proteins (Wang et al. 2020). Furthermore, reports from culture studies show that fungi assimilate about 30 to 50% of N from ferrihydrite- or goethite-associated bovine serum albumin (Wang et al. 2021). Although field studies are lacking, the ability of EM fungi to proliferate in deeper soil layers (Lindahl et al. 2007; McGuire et al. 2013; Clemmensen et al. 2021) where mineral-associated organic N is abundant (Jilling et al. 2018) and their high capacity to produce extracellular proteases (Shah et al. 2013; Nicolás et al. 2019) suggest that retrieving N from mineral-complexed proteins is important in N-limited forest ecosystems. Table 1 presents a comprehensive summary of recent findings on the abilities of distinct EM fungi to acquire N by decomposing organic substrates.

**Table 1:** Ectomycorrhizal fungal capabilities in organic matter degradation for nitrogen (N) retrieval

Fungus	N source/involved mechanisms	Measurement methods	Reference
*Paxillus involutus*	Soil organic matter/oxidative decomposition by Fenton chemistry	Infrared spectroscopy, chromatography, mass spectrometry	Rineau et al. (2012); Shah et al. (2016)
	Iron mineral-associated proteins/protein hydrolysation at the mineral surface without initial desorption	Isotopic analyses, infrared spectroscopy	Wang et al. (2020, 2021); Krumina et al. (2022)
*Suillus luteus*	Soil organic matter/oxidative decomposition by Fenton chemistry	Infrared spectroscopy, chromatography, mass spectrometry	Shah et al. (2016)
*Cortinarius* sp.	Lignin/Mn-peroxidase activity	Analysis of Mn-peroxidase activity	Bödeker et al. (2014)
*Laccaria bicolor*	Soil organic matter/oxidative decomposition	Infrared spectroscopy	Shah et al. (2016)
*Piloderma croceum*	Soil organic matter/oxidative decomposition	Infrared spectroscopy	Shah et al. (2016)
*Hebeloma cylindrosporum*	Iron mineral-associated proteins/protein hydrolysation	Isotopic analyses, infrared spectroscopy	Wang et al. (2021)
*Imleria badia*	Exogenous chitin/chitin hydrolysation	Measurements of ^15^N fungal enrichments, obtained by feeding the fungus with ^15^N labelled chitin	Maillard et al. (2023a, b)
*Boletus edulis*			
*Suillus luteus*			
*Hebeloma cylindrosporum*			

The combination of fungi’s genetic potential for organic matter degradation with their ecological strategies explains the fungal functional traits of active EM fungi in mobilising N (Maillard et al. 2023b; Auer et al. 2024). While species may exhibit similar genetic patterns of genes involved in decomposition, their expression can vary depending on fungal ecology (Barbi et al. 2016). More versatile species that readily adapt to diverse decomposing substrates may possess a high number of decomposition-related genes but exhibit lower expression levels compared to more specialised species, which may show high expression of ‘keystone functional genes’ (Barbi et al. 2016). For example, some ‘long-distance-exploration type’ EM fungi oxidise organic matter and retrieve N more effectively than some ‘short*-*’ and ‘medium-distance-exploration’ types. This is despite all of them possessing genes encoding oxidative enzymes (Shah et al. 2016). Fungal decomposition capabilities, such as enzyme activities (Courty et al. 2010; Talbot et al. 2015) and accessing N from specific sources (Chen et al. 2019), vary less among phylogenetic lineages of fungi than among species with different ecologies (Tables 1 and 2). Additionally, Op De Beeck et al. (2020) demonstrated that genetically identical hyphal tips can exhibit differences in decomposition activity at the single-cell level within the mycelium. Thus, the environmental conditions, particularly the chemical properties and the availability of the substrates, are major drivers of EM fungal traits in N mobilisation (Shah et al. 2013).

**Table 2:** Preferences of ectomycorrhizas (EM) for uptake of inorganic N from different sources

Fungus	N source preference	Methods	Reference
Beech (*Fagus sylvatica*)			
* Clavulina cristata*	NH_4_^+^	Measurements of ^15^N EM enrichments, obtained by feeding the plants with NH_4_NO_3_ labelled with either ^15^NH_4_^+^ or ^15^NO_3_^−^	Khokon et al. (2023)
* Tomentella ramosissima,*			
* Inosperma maculatum*			
* Xerocomus chrysenteron*			
* Genea hipidula*			
Helotiales	NO_3_^−^		
* Tomentella stuposa*			
* Humaria hemisphaerica*			
Douglas-fir (*Pseudotsuga menziesii*)			
* Lactarius rubrilacteus*	NH_4_^+^	Microelectrode ion flux measures	Kranabetter et al. (2015)
* Piloderma* sp.			
* Tomentella* sp.			
* Lactarius* cf. *hepaticus*			
* Lactarius luculentus*			
* Russula chloroides*			
* Tomentella sublilacina*			

## Contribution of ectomycorrhizas to inorganic N acquisition

In symbiosis, the mechanism of nutrient uptake depends on both plant and fungal nutritional status and their reciprocal influence (Sa et al. 2019; Rivera Pérez et al. 2022). Similarly to plants, EM fungi take up N from the soil in its oxidised (NO_3_^−^) and reduced (NH_4_^+^) form or as soluble organic N mono- and oligomers (Talbot and Treseder 2010; Courty et al. 2015; Garcia et al. 2016). In a direct comparison between EM and non-mycorrhizal root tips, the uptake of NH_4_^+^, measured as N fluxes at the EM mantle surface, is consistently 10 to 60 times higher in the ectomycorrhiza of various EM fungal species compared to non-mycorrhizal root tips (Hawkins and Kranabetter 2017; Hawkins and Robbins 2022). However, variation may occur in NO_3_^−^ uptake or when inorganic N availability is high (Hawkins and Kranabetter 2017; Xie et al. 2021). Nevertheless, the N fluxes measured in EM are generally higher than those in non-mycorrhizal roots (Gobert and Plassard 2002). The majority of EM fungi are particularly effective in accessing N from NH_4_^+^ (Kranabetter et al. 2015; Leberecht et al. 2016a, 2016b; Hawkins and Robbins 2022; Khokon et al. 2023), which is less mobile than NO_3_^−^ due to its adsorption onto soil cation exchange sites (Tinker and Nye 2000). This role is crucial in more acidic, cold, or poorly aerated soils where NH_4_^+^ dominates, as these conditions do not favour nitrification (Marschner 2011).

In N-rich temperate forests, such as the coastal rainforests of North America, high rates of N mineralisation may lead to elevated levels of soil inorganic N. In these ecosystems, EM fungal communities are predominantly composed of species with high NH_4_^+^ uptake capacity (e.g. *Lactarius hepaticus*, *Tomentella sublilacina*, *Tylospora* sp., Kranabetter et al. 2015). The formation of ectomycorrhiza stimulates the expression of NO_3_^−^ transporters and NH_4_^+^ transporters, as well as transporters for amino acids and peptides in both plant and fungi (Müller et al. 2020; Sun et al. 2021). While EM fungi possess only a limited number of high-affinity NO_3_^−^ transporters, they are equipped with both low and high-affinity NH_4_^+^ transporters (Garcia et al. 2016). The combination of both enables effective regulation of NH_4_^+^ uptake in response to varying soil concentrations, which likely contributes to their adaptability in N-rich environments. In the fungus, the transcriptional profile related to nutrient acquisition and transport differed between compartments. The most upregulated genes are found in the EM extraradical mycelium and the mantle, which also plays a role in nutrient storage. In contrast, the most downregulated genes, including some that are completely switched off, are observed in the Hartig net at the plant-fungal interface. This complete downregulation may represent an efficient strategy to prevent the fungal reuptake of N (e.g. ammonia, amino acids) from the apoplastic space, ensuring that N remains available for transfer to the plant (Hacquard et al. 2013; Le Tacon et al. 2015).

One of the main contributions of EM fungi to N acquisition is their ability to extend the extraradical mycelium beyond the nutrient depletion zone surrounding the roots. This extension allows them to access nutrients from a larger soil volume, compensating for the plant’s limited ability to absorb nutrients at rates faster than their loss into the surrounding soil (Pena 2016). In boreal and temperate forest ecosystems, EM mycelium comprises one-third of microbial biomass (Awad et al. 2019; Hagenbo et al. 2024). Extraradical mycelium exploration types (Agerer 2001) have long been considered to be EM traits that explain spatial foraging patterns related to resource spatial availability and acquisition (Hobbie and Agerer 2009; Zak et al. 2019). However, a recent and comprehensive study by Jörgensen et al. (2023) demonstrated that there is little support for using the external mycelium exploration type to predict EM foraging strategy. Instead, the study, along with findings by Anthony et al. (2022), found that species exhibit preferences for nutritional substrates, which can be correlated with certain degrees of hyphal hydrophobicity and nitrophobicity. Taxa with high extraradical biomass, classified as the ‘medium- and long-distance-exploration’ type, are not necessarily the most prolific. Their biomass may be sustained by a lower turnover rate (Jörgensen et al. 2023). The majority of low-proliferating taxa are nitrophobic and hydrophobic, commonly associated with inorganic N-limited environments where N acquisition from organic sources is required (Pellitier and Zak 2021; Jörgensen et al. 2023). In contrast, in a temperate forest, under relatively high atmospheric N inputs (beech forest, 13.8–16.6 kg N ha^−1^ year^−1^, Khokon et al. 2023) or in an N-rich coastal rainforest (Kranabetter et al. 2015), EM fungal communities are dominated by neutrophilic species with contact and medium smooth exploration types. Some EM fungi, in beech communities, may contribute to the uptake of NO_3_^−^, reducing NO_3_^−^ accumulation and preventing subsequent leaching (Mrak et al. 2024). Nevertheless, the species within these communities exhibited significant variation in N uptake of NH_4_^+^ or NO_3_^−^ (Khokon et al. 2023), corroborating previous findings of substantial variability in fungal abilities for N acquisition. Table 2 highlights the diversity among ectomycorrhizas in N acquisition from various sources.

A recent metatranscriptomic study revealed that the impact of the EM community on tree N nutrition was similar and consistent across different fungal communities despite being composed of different taxa. The authors have suggested that functional redundancy exists among ectomycorrhizas (Auer et al. 2024). Evidence from other studies also suggests that communities, assembled through environmental filtering, are dominated by species best equipped to utilise the most available N source in their environment (Kranabetter et al. 2015). Moreover, endemic EM fungi are better adapted than cosmopolitan species at exploiting available N sources, indicating a high level of specialisation in enhancing tree access to available N (McPolin et al. 2024). On a global scale, the composition of EM fungal communities has been shown to account for a threefold variation in tree growth (Anthony et al. 2022). In contrast, Khokon et al. (2023) found that the positive relationship between EM fungal diversity and tree N acquisition does not correlate with any particular taxa, suggesting that specific traits of fungal species do not solely explain root N acquisition. This indicates that ectomycorrhizal communities contribute to tree N nutrition in a species-specific but complementary manner. A high EM functional diversity in abilities to access distinct or spatially scattered N sources forms the basis for improved N acquisition. We may consider physiological and functional flexibility, even within a single mycelial network, and adaptability to the edaphic environment to be within the limits of the reciprocal invested benefits (Cairney and Burke 1996). For example, in an EM assemblage, distinct EM taxa activate their N uptake abilities to benefit the tree when abiotic conditions are limiting, but not when the tree is unstressed (Pena and Polle 2014). Sustaining high EM fungal biodiversity is critical for tree N nutrition under current and future climate scenarios. However, maintaining the reciprocal investments and benefits of mutualistic interactions has sensitive limits. As EM fungal community size increases, this sensitivity may also rise, potentially leading to pseudo-reciprocity, appropriated benefits, or parasitism.

## C and N resources in the ectomycorrhizas

The tree plays an intrinsic role in EM-mediated N nutrition, either by decoupling its N metabolism from fungal metabolism (Leberecht et al. 2016a; Rivera Pérez et al. 2022), or by modulating C allocation to ectomycorrhizas. The maintenance of diverse EM communities depends on the tree’s C supply (Pena et al. 2010). Furthermore, EM fungi that receive more C can colonise more root tips compared to those receiving less carbon (Pena et al. 2023). In other nutritional symbioses, such as the arbuscular mycorrhizal (Kiers et al. 2011) or legume–rhizobium (Simms et al. 2006) symbioses, the nutrient flux between partners follows the market exchange theory, with the plant allocating more C to the partner that provides the most nutrients. In ectomycorrhizas, at the cellular scale, the fungal-acquired soil N is spatially correlated with the plant photo-assimilated C transferred to the fungus (Mayerhofer et al. 2021). However, there is no quantitative correlation between the two fluxes (Valtanen et al. 2014; Hortal et al. 2017; Plett et al. 2024). Nevertheless, a strong relationship exists between the C supply and the taxon-specific C/N ratio of ectomycorrhiza, with the C supply decreasing as the C/N ratio increases. No C supply occurs when C/N is high (C/N > 24, Pena et al. 2023). This indicates that the plant-fungus exchange is not linear. The plant controls C allocation based on N content of the ectomycorrhiza, while fungal traits for N use efficiency, which determine N uptake and immobilisation in the fungus, provide feedback control on plant C allocation (Pena et al. 2023). EM feedback probably varies depending on environmental conditions and nutrient availability. A critical situation can arise under N-limitation when EM fungi take up and immobilise N in their biomass without supplying it to the trees, while continuously receiving C from them. Fungal-supplied N is correlated to the concentration of free amino acids in EM extraradical mycelium (Plett et al. 2024). In boreal forests, several studies suggest that high C allocation to symbionts enables greater fungal N immobilisation, negatively affecting soil N availability and forest N cycling (Hasselquist et al. 2016; Högberg et al. 2017; Henriksson et al. 2021).

According to Pena et al. (2023), different ectomycorrhizas form distinct plant-C sinks depending on their C/N ratio but also represent species-specific sinks of plant-assimilated N. In angiosperm plants, N absorbed from the soil is primarily assimilated into amino acids in the leaves. These amino acids are then redistributed to developing organs via the phloem, serving as the primary N source for root growth (Yoneyama et al. 2003). Internal N availability regulates tree N uptake (Rennenberg and Dannenmann 2015) and influences tree N-acquisition strategies, such as root proliferation to exploit soil N hotspots (Chen et al. 2018). In a recent study, using secondary ion mass spectrometry (SIMS) imaging combined with leaf labelling of young beech with ^15^NH_4_^+^, plant-derived N was found to be present in the fungal tissue within ectomycorrhizas (Pena et al. 2023). The ^15^N enrichment in the lateral rootlets was also correlated with the enrichment found in the attached ectomycorrhizas, which was further correlated with their C/N ratio. Ectomycorrhizal fungi can capture the plant-assimilated N either from the root apoplast at the symbiotic interface or by recapturing it after exudation. In the first scenario, EM fungal intervention is less likely because the presence of fungal amino acid transporters at the symbiotic interface could intercept the N influx, destabilising the symbiosis functionality (Martin & Nehls 2009, but see Garcia et al. 2016). Nevertheless, the second scenario is more probable, as EM fungi can uptake amino acids from the soil (Garcia et al. 2016). Given that the fungal mantle tightly encapsulates the EM root tip, EM fungi are favoured over other soil microorganisms in accessing plant-exuded N (Canarini et al. 2019). By intercepting the root N efflux, EM fungi reduce the supplementary N source available for rhizosphere microorganisms (Jones et al. 2004; Canarini et al. 2019). This functional trait of EM fungi, either creating a distinct sink for plant-assimilated N or recapturing the plant-exuded N, is crucial for tree N nutrition. It provides the basis for EM-regulated N fluxes within the root system and directly affects the plant’s priming capacity by modifying the exudate C/N stoichiometry. This latter aspect is important, as microorganism activity is commonly constrained by both C and N availability (Jones et al. 2004; Drake et al. 2013).

## Ectomycorrhizal fungi enhance tree N nutrition via microbiome influence

The role of EM fungi in plant nutrition also includes an indirect component, as they positively influence other soil microorganisms (e.g. bacteria, archaea) involved in N cycling, thereby enhancing plant N uptake (Frey-Klett et al. 2007; Lladó et al. 2017; Uroz et al. 2019). Mycorrhizal symbiosis creates new niches for microorganisms by modifying the plant’s ecophysiological traits and local soil properties (Uroz et al. 2019). Specifically, EM fungi construct a unique compartment of the mycorrhizosphere—the immediate space surrounding the external EM hyphae influenced by root and hyphal exudates. This compartment provides space and nutrients for a range of microbial communities (Johansson et al. 2009; Bogar and Peay 2017; Gorka et al. 2019). For example, the mycorrhizospheres of *Pinus sylvestris* (Rinta-Kanto and Timonen 2020) and *Pinus muricata* (Nguyen and Bruns 2015) are populated with Actinobacteria and Planctomycetia, which can break down recalcitrant organic substrates (e.g. chitin) to retrieve N, and Burkholderiales, which are involved in N fixation (Elliott et al. 2007).

A much deeper analysis of *Pinus sylvestris* (Marupakula et al. 2016) or *Fagus sylvatica* (Dietrich et al. 2022) EM root tips revealed that distinct EM fungi harbour distinct communities of bacteria. Furthermore, rare fungal taxa play a role similar to that of the most abundant taxa in driving the assembly of new microbial communities (Dietrich et al. 2022). These findings highlight the importance of maintaining a high mycorrhizal diversity. High functional diversity is essential to foster the formation of diverse associated microbial communities that enhance N cycling and plant N nutrition. Further research is needed to understand how the functional benefits of EM fungi can remain unaffected by environmental changes and disturbances, ensuring sustained plant growth and soil health.

## Future perspectives

Understanding the dynamics of EM functions along the mutualism-parasitism continuum in forest ecosystems is essential for the effective management of ecosystem restoration and resilience amidst climate change. Variation in mutualistic species interactions is common in nature, particularly in mycorrhizal symbiosis, which involves a bidirectional energy transfer –C from plants to fungi and N or other nutrients from fungi to plants. This variation is often described as context-dependent, influenced by changes in biotic factors, such as the involvement of additional species, and abiotic factors, such as resource availability or abiotic stress (Chamberlain et al. 2014) (Fig. 1b).

In the biotic context, the immediate functional groups that may influence EM symbioses through competition or cooperation with EM fungi include other fungi that occupy the same spatial niche and can transfer N to plants and access plant C resources. Tree roots host rich communities of dark septate endophytic (DSE) fungi that live in plant tissues, producing no symptoms or morphological modifications of their hosts (Hardoim et al. 2015). These ascomycetes have a strong enzymatic potential, enabling them to acquire N from organic sources, which is then transferred to the tree. There is a by-product mutualist interaction in which DSEs provide N without requiring any special structural investment from the host plant (Ruotsalainen et al. 2022). Tree C, which enters the soil as root exudates, or leaf and root litter, is a by-product benefitting the DSE fungi (Ruotsalainen et al. 2022). This interaction is considered a transitional phase in the evolution of mycorrhizal symbionts from saprotrophic fungi (Ruotsalainen et al. 2022). Reports on the interaction between DSE and EM fungi are currently limited and exhibit significant variability, ranging from neutral to competitive or facilitative interactions, largely depending on the fungal strains involved (Reininger and Sieber 2012; Berthelot et al. 2019). Notably, these findings are derived from in vitro experiments (Berthelot et al. 2019) and growth chamber studies with seedlings (Reininger and Sieber 2012), with no data available from field studies. Future research should prioritise field investigations to better understand the effects of DSEs on EM symbioses in natural settings and their implications for tree nutrition.

A special type of root endophyte involved in plant N acquisition is the soil ascomycetes fungi, such as *Beauveria* sp. (Cordycipitaceae) and *Metarhizium* sp. (Clavicipitaceae), which function as both endophyte and insect pathogens and can acquire N from soil insects and transfer it to the plant in exchange for C (Hu and Bidochka 2021; Bamisile et al. 2023). Their role in plant N nutrition was first described in 2012 (Behie et al. 2012). However, there are no reports on their interaction with other root fungi or whether their N-transfer abilities might affect the effectiveness and stability of mycorrhizal symbioses, which also deliver N to the plants.

Apart from fungi that reside in the same root with EM fungi, there are also feremycorrhizal fungi, meaning ‘nearly mycorrhizal.’ These fungi exhibit traits and functions similar to those of EM fungi but do not penetrate the roots (Kariman et al. 2014). Unlike EM symbiosis, no investment from the plant is required. Thus, feremycorrhiza is a by-product interaction that may interfere with mycorrhizas, potentially affecting their mutualistic relationship with the host plant.

In the abiotic context, EM mutualism can be influenced by current changes in soil nutrient levels. Historically, N has been a limiting factor in forest ecosystems of the Northern Hemisphere. However, due to anthropogenic activities, the global availability of N has increased to unprecedented levels, disrupting the context of low N supply and tight recycling (Galloway et al. 2008). Along natural fertility gradients, in boreal forests, increased N availability may have a positive effect on mycelium growth and species richness (Kranabetter et al. 2009a, 2009b; Högberg et al. 2021). In temperate N-rich forests, EM fungal communities remain diverse, with no apparent decline in diversity observed even under conditions of extreme native soil fertility compared to less fertile environments (Kranabetter et al. 2015). A recent study conducted in boreal forests found that moderate N deposition (5.8 kg N ha^−1^ year^−1^) had no impact on EM fungal biomass and community composition (Jörgensen et al. 2024). However, more severe N deposition (e.g. 11.1 kg N ha^−1^ year^−1^, Jörgensen et al. 2024) may lead to declines in fungal sporocarps, biomass, abundance, and community diversity. EM fungal communities tend to shift from nitrophobic taxa under moderate N deposition (5.8 kg N ha^−1^ year^−1^) to nitrophilic taxa at higher levels (15.5 kg N ha^−1^ year^−1^, van der Linde et al. 2018). This shift includes the loss of key functional species, particularly those with high enzymatic capabilities for releasing N from organic sources (reviewed by Lilleskov et al. 2019, 2024). Currently, it remains unclear whether the effects of N deposition on EM fungi differ between communities that are already more nitrophilic due to adaptation to N-rich soils and those from low-N environments. These observations related to N deposition are likely driven by either direct N toxicity stress or by alterations in EM symbiosis. This symbiosis is fundamentally based on the plant’s need for limited nutrients under an invested benefits mutualism. With no N limitation, the symbiosis may become a by-product benefit when plant C supply to fungi is at no expense for the plant or an appropriated benefit when the C supply is costly for the plant.

Changes in soil nutrient stoichiometry, such as P limitation induced by N saturation (Sardans et al. 2016), trigger changes from an N to a P-oriented acquisition strategy mediated by roots and ectomycorrhizas (Meeds et al. 2021; Zhang et al. 2023; Zhu et al. 2023). Averill et al. (2018), using a Bayesian multiple regression framework, found that across the USA, N deposition is linked to a decline in EM tree species, favouring their replacement with arbuscular mycorrhizal tree species. This shift is commonly explained by the fact that arbuscular mycorrhizal fungi primarily rely on inorganic N forms and possess significant abilities in P acquisition.

However, a recent finer-scale metanalysis in the tropics showed that the distribution and abundance of EM and arbuscular mycorrhizal trees are independent of soil nutrient availability (Medina-Vega et al. 2024). The results were supported by an empirical study of Chilian native forests (Lusk et al. 2024). At a plant level, research involving dual plants, capable of forming both arbuscular and EM symbioses, has revealed a certain plasticity in root symbioses to optimise nutrient acquisition under P limitation. However, a direct switch from EM to arbuscular mycorrhizal symbiosis was not apparent (Teste and Laliberté 2019). Under a higher N/P ratio and reduced pressure for N acquisition, EM fungi may benefit from the plant’s ability to allocate more energy toward increasing organic P acquisition through EM fungi or other mechanisms (McPolin et al. 2024), such as enhancing the activity of enzymes involved in P acquisition (Meeds et al. 2021).

Forests cover much of the Earth’s surface, providing crucial ecosystem services. With anthropogenic changes in temperature, precipitation, and N deposition, it is vital to understand the factors influencing the EM nutrient exchange and interactions with soil organisms affecting N supply to plants. Changes in plant-fungal mutualism within ectomycorrhizal symbiosis and reduced EM functional diversity are critical for forest productivity, soil carbon sequestration, nutrient cycling, and climate change feedback. Future studies should identify the factors and mechanisms driving these changes to mitigate their impacts and preserve forest ecosystem services.

## Data Availability

This review paper does not contain associated laboratory data.
